# Changes in best-corrected visual acuity in patients with dry age-related macular degeneration after stem cell transplantation: systematic review and meta-analysis

**DOI:** 10.1186/s13287-022-02931-y

**Published:** 2022-06-07

**Authors:** Licheng Li, Yang Yu, Shu Lin, Jianmin Hu

**Affiliations:** 1 Department of Ophthalmology, The Second Affiliated Hospital of Fujian Medical University, Engineering Research Centre of Assistive Technology for Visual Impairment, Fujian Province University, No. 34 North Zhongshan Road, Quanzhou, 362000 Fujian Province China; 2 Centre of Neurological and Metabolic Research, the Second Affiliated Hospital of Fujian Medical University, No. 34 North Zhongshan Road, Quanzhou, 362000 Fujian Province China; 3 Group of Neuroendocrinology, Garvan Institute of Medical Research, 384 Victoria St, Sydney, Australia; 4 The School of Medical Technology and Engineering, Fujian Medical University, Fuzhou, Fujian Province China

**Keywords:** Meta-analysis, Stem cell transplantation, Dry age-related macular degeneration

## Abstract

**Background:**

Stem cell transplantation may improve visual acuity in patients with dry age-related macular degeneration. Herein, we aimed to summarise the evidence on the risks and benefits of stem cell transplantation for improving visual acuity, including the risk of adverse events.

**Methods:**

Data were obtained from the PubMed, EMBASE, and the Cochrane Central Register of Controlled Trials databases, and each database was interrogated from the date of inception until 19 March 2022. The rates of visual acuity outcomes and adverse events associated with stem cell transplantation were examined. All statistical analyses were conducted using Review Manager 5.4. The study was registered with PROSPERO (CRD 42022322902).

**Results:**

The analysis examined 10 studies (102 patients), including one and three, randomised and non-randomised clinical trials, and one and five, multicentre prospective and prospective clinical trials, respectively. Meta-analysis showed changes in best-corrected visual acuity in the study eyes after stem cell transplantation (6 months: risk ratio [RR] = 17.00, 95% confidence interval [CI] 6.08–47.56, *P* < 0.00001; 12 months: RR = 11.00, 95% CI 2.36–51.36, *P* = 0.002). Subgroup analysis showed that different stem cell types achieved better best-corrected visual acuity at post-operative 6 months, compared to that observed at baseline. Four cases of related ocular adverse events and no related systemic adverse events were reported.

**Conclusion:**

This meta-analysis suggests that stem cell transplantation may improve best-corrected visual acuity in dry age-related macular degeneration, based on small sample sizes and fewer randomised controlled trials.

**Supplementary Information:**

The online version contains supplementary material available at 10.1186/s13287-022-02931-y.

## Background

**Fig. 1:**
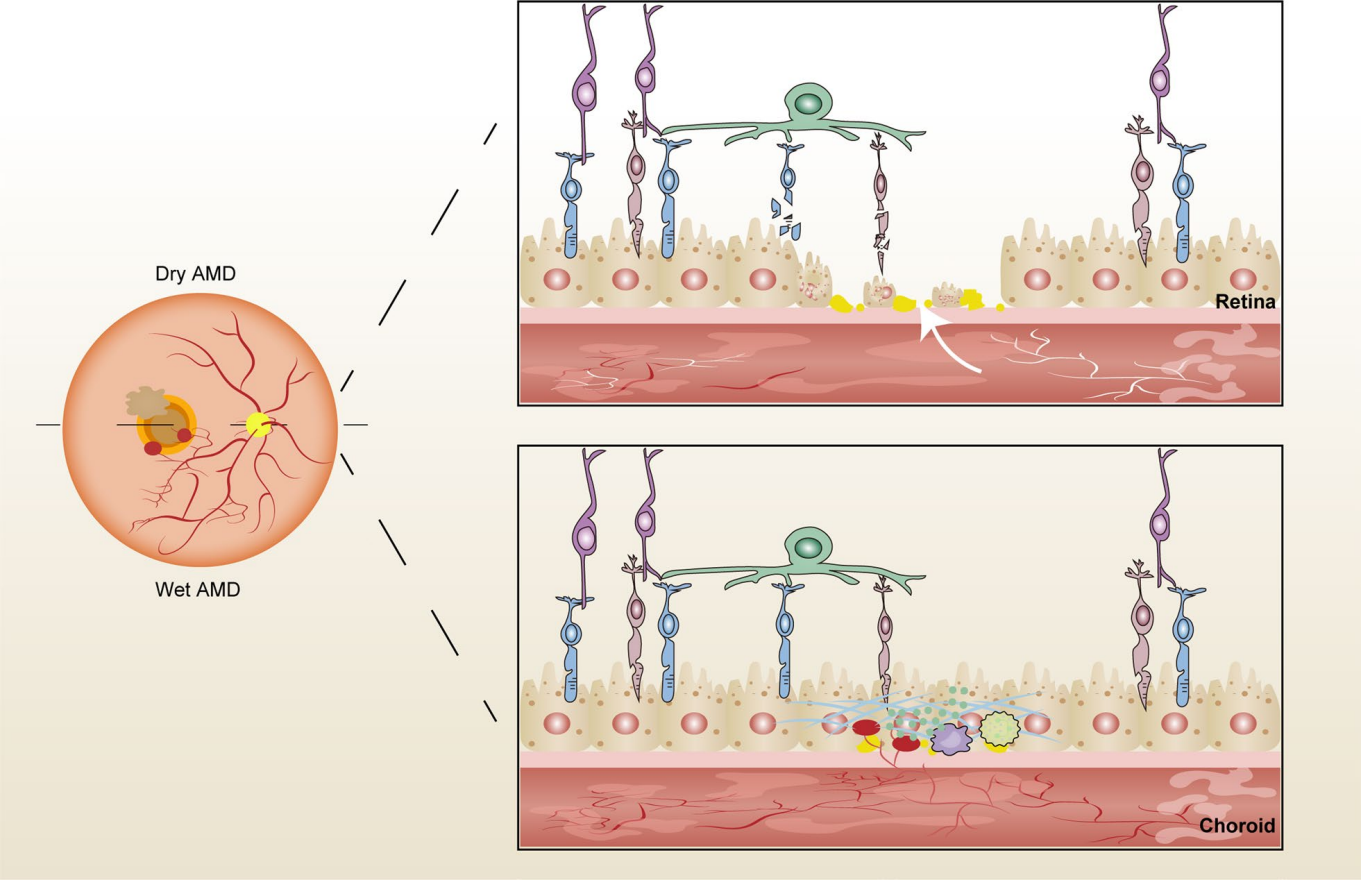
Schematic of the underlying mechanisms in advanced AMD. Several factors affect extracellular deposits in the retina. Dry AMD is caused by progressive thinning of the central retina, reduced nutrient supply from the thinned choriocapillaris, atrophic RPE with degrading and dying photoreceptors, and non-functional area formation. Wet AMD is caused by inflammation and abundant complement. Subsequent pathological angiogenic stimulus leads to neovascularisation, serum-haemorrhagic exudation, and fibro-scarring under the neuro-epithelium and in the retinal stroma

Age-related macular degeneration (AMD) is an ophthalmic disease that causes progressive damage to the macula, leading to irreversible vision loss and blindness [1]. In 2020, AMD was estimated to affect 196 million people globally, incurring significant costs at individual and societal levels [2]. Approximately 288 million people worldwide will be affected by AMD by 2040 [2]. AMD is characterised by the accumulation of extracellular deposits, known as drusen, accompanied by the progressive degeneration of photoreceptors and adjacent tissues [3]. Disease progression is associated with chronic inflammation, retinal cell metabolic rate reduction, oxidative stress, and extracellular matrix reduction, resulting in a gradual and progressive degeneration and the loss of the retinal pigment epithelium (RPE) cells initially, followed by that of photoreceptors and adjacent tissues [4]. Over time, if inflammation persists and complement levels increase, neovascular damage results in fluid leakage or haemorrhage from the highly permeable subretinal or sub-RPE vascular networks [4]. Multiple factors (ageing, diet, smoking, genetics, and other environmental factors) increase the risk of AMD [5]. According to the epidemiological classification, AMD is classified into the early, intermediate, and advanced (atrophic/dry/non-vascular AMD or exudative/wet/neovascular AMD) [6]. In fact, 85–90% of dry AMD cases are associated with several genetic factors [7] and are caused by the progressive thinning of the central retina, reduced nutrient flow from the thinned choriocapillaris, an atrophic RPE with subsequent degradation and death of photoreceptors, and the formation of non-functional areas that resemble “geographical maps” [8]. Best-corrected visual acuity (BCVA) tends to decrease gradually but inexorably [9]. In contrast, 10–15% of wet AMD cases are associated with genetic changes that are distinct from those implicated in dry AMD [7]. The combination of inflammation and abundant complement is a pathological angiogenic stimulus that leads to neovascularisation, serum-haemorrhagic exudation, and fibro-scarring under the neuro-epithelium and in the retinal stroma, and triggers vision loss [4] (Fig. 1).

Dietary supplementation of formulations with antioxidant, haemorheological, and anti-inflammatory properties is recommended. In general, the functional improvement is modest and short term, but these formulations may slow down disease progression in the early and intermediate stages [10, 11]. Previously, photodynamic therapy was used for the treatment of wet AMD; however, the current approach to treatment involves monoclonal antibodies that act on vascular endothelial growth factor (VEGF) receptors [10, 12, 13], and which are administered via an intravitreal injection. However, anti-VEGF therapy does not counteract the underlying atrophic mechanisms, and AMD continues to progress [14]. There is currently no effective treatment for dry AMD [10]. Therefore, owing to the genetic, pathological, and clinical differences between dry and wet AMD, we focussed only on the dry form in this meta-analysis.

Stem cell transplantation (SCT) is a promising treatment for dry AMD. Stem cells can be derived from the embryonic, reprogrammed, or autologous tissues. Essentially of autologous tissues is mesenchymal origin which can be divided into two subgroups, including bone marrow and adipose tissues. Several preclinical trials have shown that transplanted stem cells survive and retain some functionality in C57BL/6 J mice and rabbits with retinal degeneration [15, 16]. In rats with retinal degeneration, the induced pluripotent stem cell-derived retinal pigment epithelium (iPSCs-RPE) was integrated into the retina and maintained phagocytic function for 2.5 years after transplantation without any adverse events [17]. Stem cells may preserve or promote the survival and function of diseased cells by expressing suitable secretomes, including cytokines and exosomes released via the paracrine system [18]. The mesenchymal secretome may have haemorheological, antioxidant, anti-inflammatory, anti-apoptotic, and neuro-protective properties, which promote the survival and functional restoration of the retinal cells. Findings from preclinical studies are encouraging, in particular, for mesenchymal cells. Some studies have examined repair processes associated with ganglion structures in glaucomatous opticopathy [19–22], and those associated with photoreceptor structures in Stargardt disease and dry AMD [23, 24], as well as in retinitis pigmentosa (RP). Cell therapy or cell-mediated therapy may be defined as any therapeutic modality based on the use of cell transplants or grafts that aims to neuro-enhance compromised cells, supporting repair or regeneration, including replacement, of receptors, mitochondrial components, and connecting fibres, while integrating with the remaining retinal structures [25, 26]. In 2014, a 70-year-old Japanese woman with AMD became the first person in the world to receive treatment based on iPSCs [27]. Subsequently, two patients with advanced AMD received the iPSCs-RPE transplantation and the transplanted sheet remained intact after 1 year [28]. Recently, clinical trials have shown that SCT can improve visual acuity in patients with AMD. However, some studies have also reported retinal detachment (RD) after the surgery in patients with AMD [29, 30].

No previous systematic review or meta-analysis has assessed the rates of visual acuity outcomes and those of adverse events in patients with dry AMD undergoing SCT. This systematic review and meta-analysis aimed to summarise the current evidence on these outcomes.

## Methods

The systematic review protocol was registered on the International Prospective Register of Systematic Reviews (PROSPERO) website (CRD 42022322902). Moreover, this meta-analysis followed the updated guidelines for the reporting of systematic reviews (PRISMA 2020 statement) [31].

### Search strategy

We searched the PubMed, EMBASE, and the Cochrane Central Register of Controlled Trials databases for studies on macular degeneration and stem cell transplantation using Medical Subject Headings and free words from database inception until 19 March 2022 (Additional file 1: Table S1). No ethical approval was required for this study because the meta-analysis was based on published articles.

### Eligibility criteria

Studies were included according to the following criteria: (a) patients diagnosed with advanced AMD; (b) patients aged ≥ 50 years; and (c) eyes treated with SCT. Studies were excluded if they involved patients with prior or current choroidal neovascularisation or any evidence of neovascular AMD in either eye. Relevant case reports were excluded.

To examine the impact of SCT on BCVA in dry AMD, patients were divided into preoperative and post-operative groups.

### Data collection

Duplicate records were removed using EndNote X9 (Clarivate Analytics, Philadelphia, PA, USA). Two authors (LL and YY) screened titles and abstracts to identify potentially eligible articles and then read the full text before study inclusion. For multiple studies based on the same dataset, only the most recent publication was included. Two reviewers independently extracted data on study (author, year, country, study design, patient, and follow-up) and patient (sex, age, diagnosis, stem cells, and administration routes) characteristics. Any discrepancies were resolved by arbitration by a third reviewer (SL).

### Quality assessment

The risk of bias in randomised studies was assessed with the Cochrane Collaboration tool, based on: allocation sequence generation, allocation concealment, masking by patients and clinicians, masking by outcome assessors, incomplete outcome data, selective outcome reporting, and other sources of bias. Moreover, we used the Cochrane risk of bias tool for non-randomised studies of interventions [32]. This tool accounts for confounding, selection, classification, deviations from intended interventions, missing data, outcome measurement, and reported result selection.

### Effect measures

To compare BCVA before and after SCT, the results were aggregated and presented using risk ratios. Adverse event rates were reported, as relevant.

### Statistical analysis

All data analyses were performed using the Review Manager (version 5.4; Cochrane Collaboration). We analysed changes in BCVA after SCT in the study eyes. Heterogeneity was quantified using *Q*, *H*, and *I*^2^ statistics. We performed meta-analysis using a fixed-effects model. If heterogeneity was high (*I*^2^ > 50%), we used a random-effects model. Subgroup analysis was performed to investigate the effects of different stem cell types. Sensitivity analyses were conducted by removing individual studies. A funnel plot was drawn to explore publication bias.

## Results

**Fig. 2:**
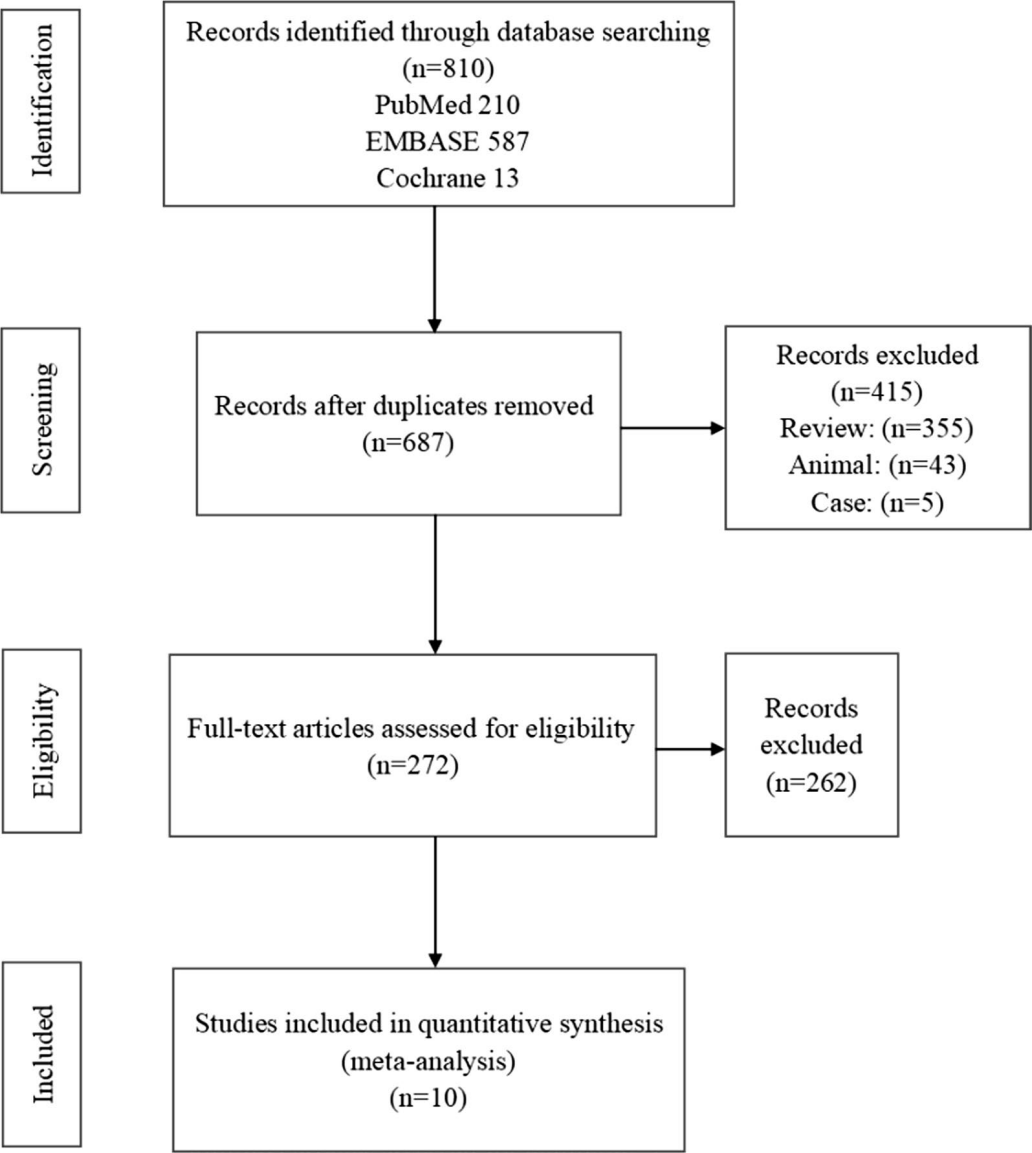
Flow chart of the selection of clinical studies on SCT therapy for dry AMD. In total, 810 were identified in the database search (210 in PubMed, 587 in EMBASE, and 13 in Cochrane). Duplicate records, reviews, animal studies, case reports, and unrelated articles were removed. Ten clinical studies were included in the meta-analysis

The search identified 810 articles (210 in PubMed, 587 in EMBASE, and 13 in Cochrane). Ten unique clinical studies [23, 33–41] were included in data analysis (Fig. 2). Three articles [34, 42, 43] were based on the same dataset; only the latest article [34] was included. Among these studies, one [36] was a randomised prospective clinical trial, three [34, 35, 40] were non-randomised prospective clinical trials, one [38] was a multicentre prospective clinical trial, and five [23, 33, 37, 39, 41] were prospective clinical trials. In total, 102 patients with dry AMD who underwent SCT were included in this meta-analysis. The age range of most patients was 59–88 years (Table 1).

**Table 1:** Included study and patient characteristics

Author	Year	Country	Study design	Patients	Sex (M/F)	Age (years)	Diagnosis	Stem cells	Administration routes	Follow-up (mth)
Nittala [38]	2021	USA	Multicentre PCT	15	–	–	Dry AMD	HuCNS-SCs	Subretinal	12
Kashani [34]	2021	USA	Non-randomised PCT	16	7/9	69–85	Dry AMD	hESC-RPE	Subretinal	12
Oner [23]	2018	Turkey	PCT	8	5/3	19–75	Dry AMD or SMD	ADSCs	Suprachoroidal	6
Limoli [36]	2018	Italy	Randomised PCT	25	14/11	62–84	Dry AMD	ADSCs	Suprachoroidal	6
Cotrim [33]	2017	Brazil	PCT	10	–	–	Dry AMD	BM with CD34 + SCs	Intravitreal	12
Limoli [35]	2016	Italy	Non-randomised PCT	25	9/16	64–84	Dry AMD	ADSCs	Suprachoroidal	6
Song [41]	2015	Republic of Korea	PCT	4	–	–	Dry AMD or SMD	hESC-RPE	Subretinal	12
Schwartz [40]	2015	USA	Non-randomised PCT	18	–	20–88	Dry AMD or SMD	hESC-RPE	Subretinal	12
Park [39]	2014	USA	PCT	6	4/2	23–85	Dry AMD or SMD or RP or CRAO	BM with CD34 + SCs	Intravitreal	6
Limoli [37]	2014	Italy	PCT	12	6/6	62–80	Dry AMD	ADSCs	Suprachoroidal	1

CRAO, central retinal artery occlusion; PCT, prospective clinical trial

### Risk of bias assessment

**Fig. 3:**
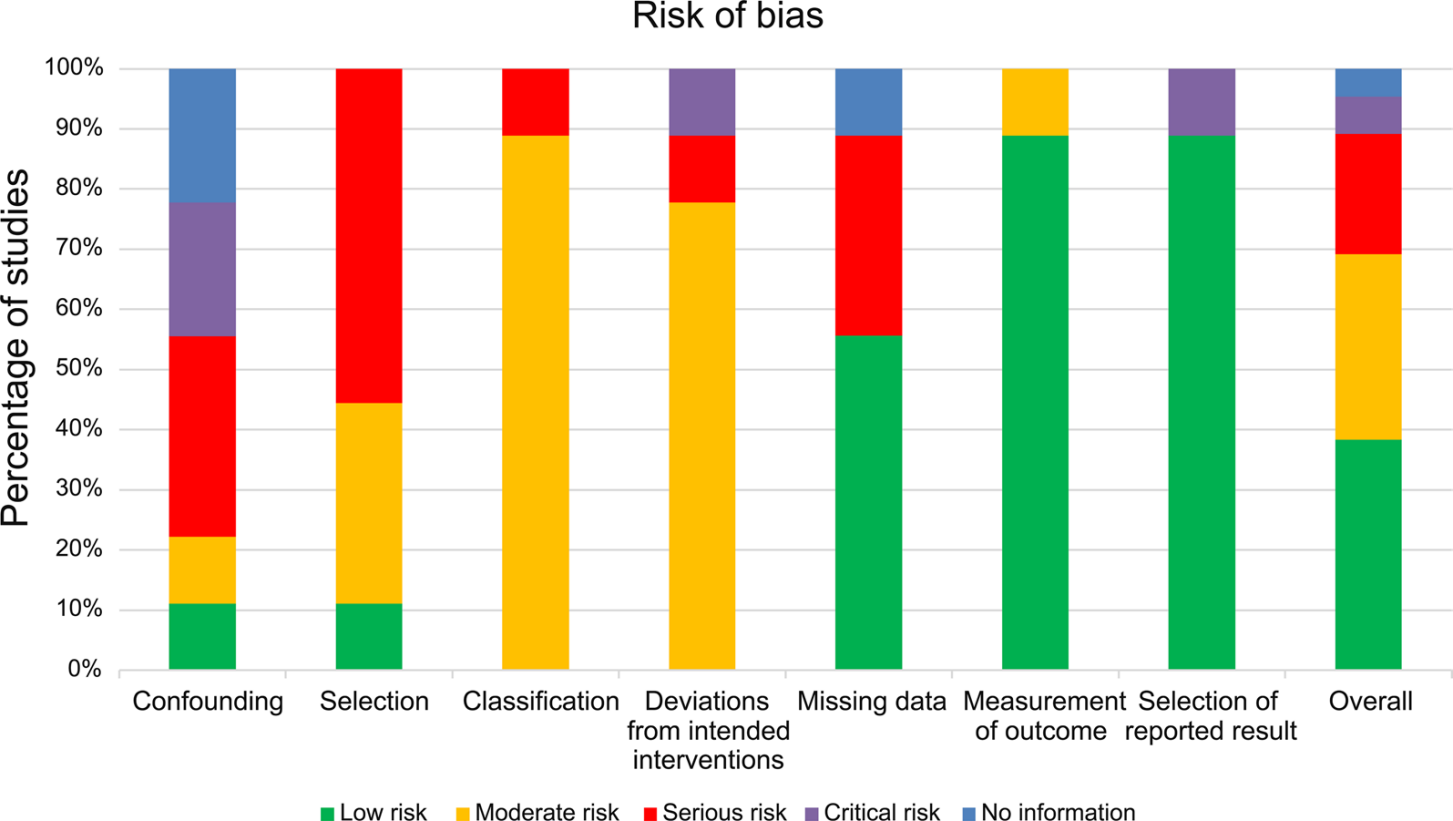
Risk of bias summary for non-randomised studies. Among nine non-randomised clinical studies, six studies reported confounding factors. Eight studies were at a risk of selection bias. Nine studies were at a risk of bias from classification and deviations from intended interventions. Four studies were at a risk of bias from missing data. One study was at a risk of bias from outcome measurement and result reporting

**Fig. 4:**
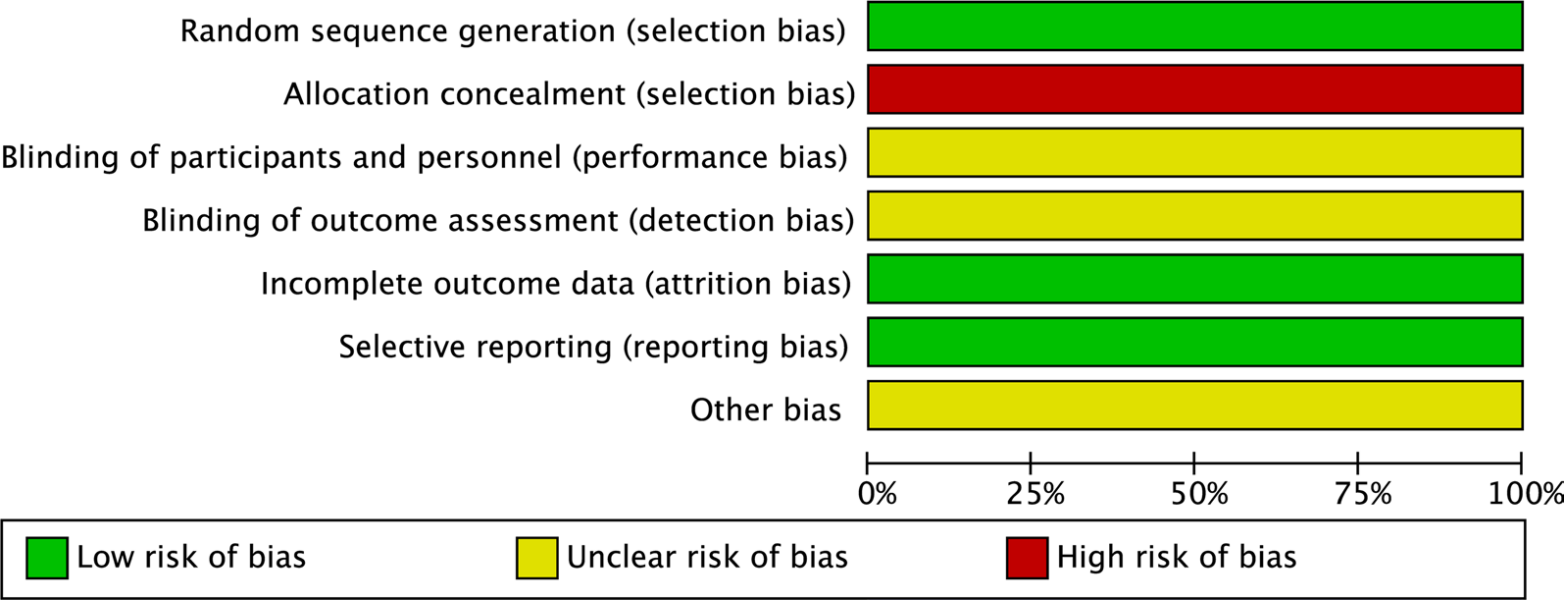
Risk of bias summary for a randomised study. One randomised clinical study was at a high risk of bias from allocation concealment, and at a low risk of bias from random sequence generation, incomplete outcome data, selective reporting, and at unclear risks of bias from performance, detection, and other sources

The risk of bias findings for non-randomised and randomised clinical studies are presented in Figs. 3 and 4, respectively. Among nine non-randomised clinical studies, six studies were affected by baseline and time-varying confounding. Eight studies were affected by selection bias, whereby most of the included AMD patients had BCVA of ≤ 40/200. Nine studies were at a risk of bias from the classification of interventions and deviations from intended interventions. Four studies were at a risk of bias due to missing data. Four studies were at a risk of bias due to the loss to follow-up. One study was at a risk of bias from the measurement of outcomes and selection of the reported results. Moreover, one randomised clinical study was at a high risk of bias in allocation concealment because patients knew which eye was receiving stem cell-based therapy. The risks of bias from performance, detection, and other sources were unclear. The remaining bias was at a low risk.  were Overall, the methodological quality of the included studies was acceptable.

### Best-corrected visual acuity

**Fig. 5:**
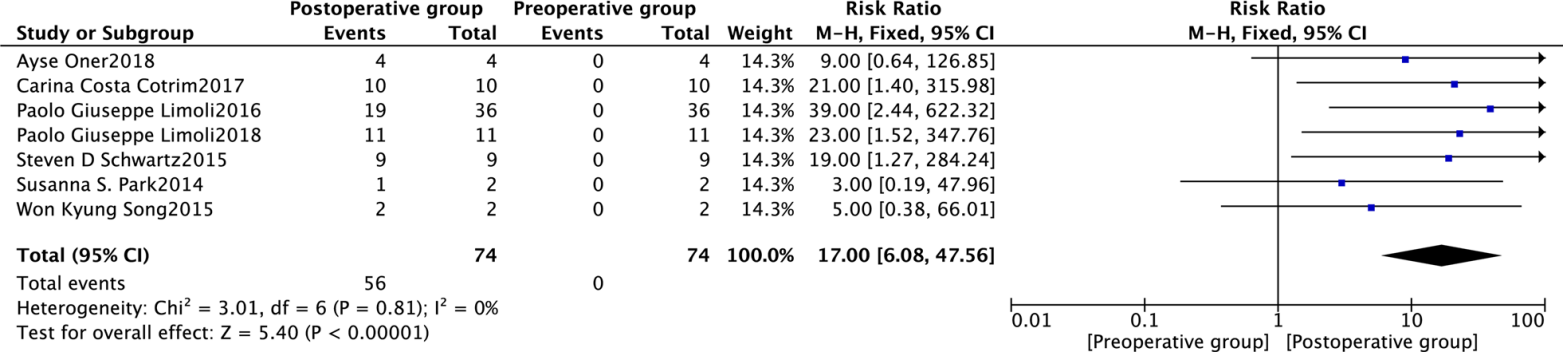
Forest plot showing the impact of SCT therapy on BCVA at month 6. The risk ratio in the fixed-effects model between post-operative and preoperative groups in BCVA at month 6 was 17.00 (95% confidence interval: 6.08–47.56, *P* < 0.00001). A low degree of heterogeneity was observed (*I*^2^ = 0%). The post-operative group at month 6 was favoured

**Fig. 6:**
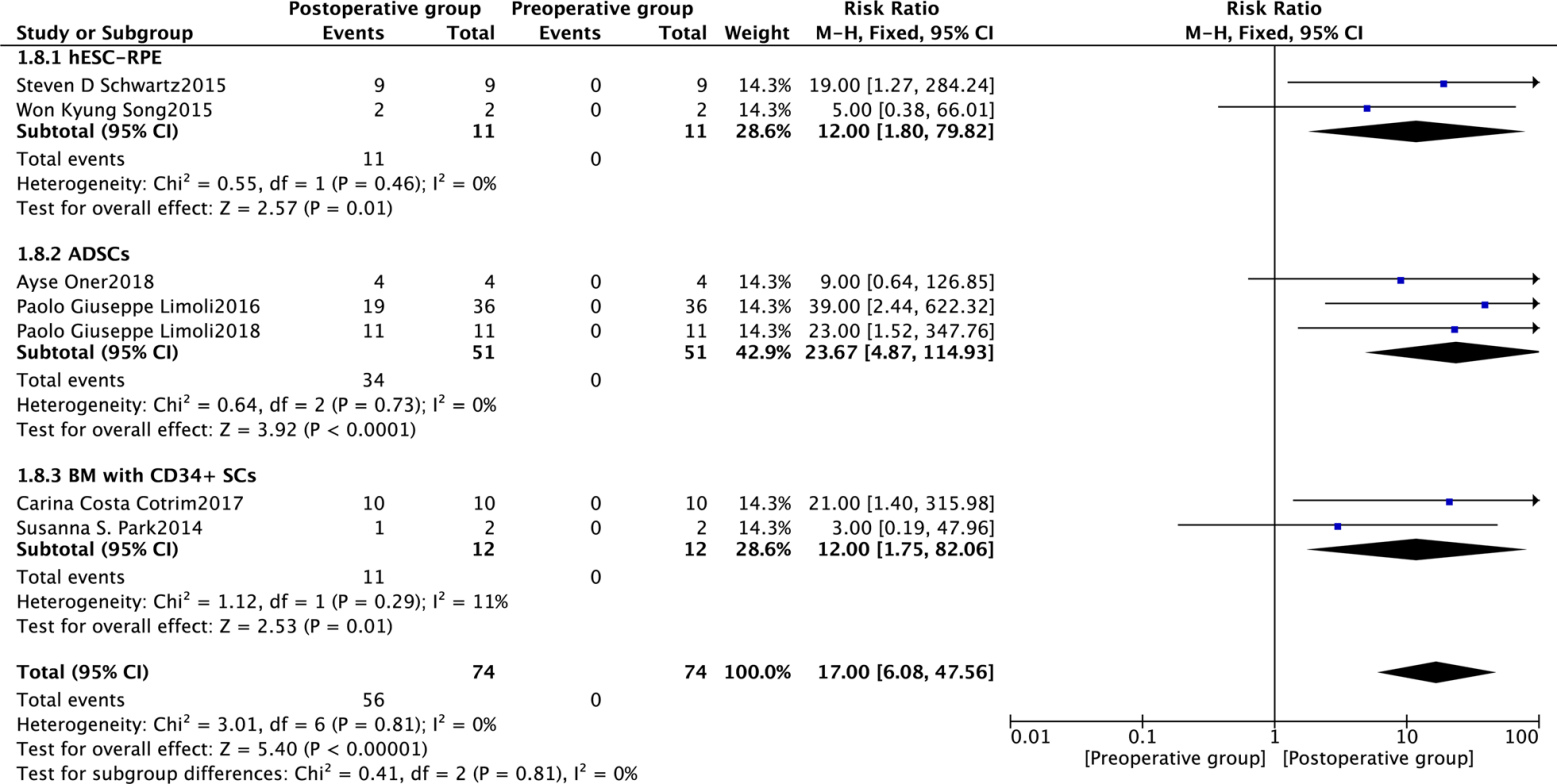
Subgroup analysis showing the effect of different stem cells on BCVA at month 6. The risk ratio of hESC-RPE in the fixed-effects model between post-operative and preoperative groups in BCVA after 6 months was 12.00 (95% confidence interval [CI] 1.80–79.82, *P* = 0.01). The risk ratio of ADSCs in the fixed-effects model between post-operative and preoperative groups in BCVA after 6 months was 23.67 (95% CI 4.87–114.93, *P* < 0.0001). The risk ratio of BM with CD34 + SCs in the fixed-effects model between post-operative and preoperative groups in BCVA after 6 months was 12.00 (95% CI 1.75–82.06, *P* = 0.01)

**Fig. 7:**
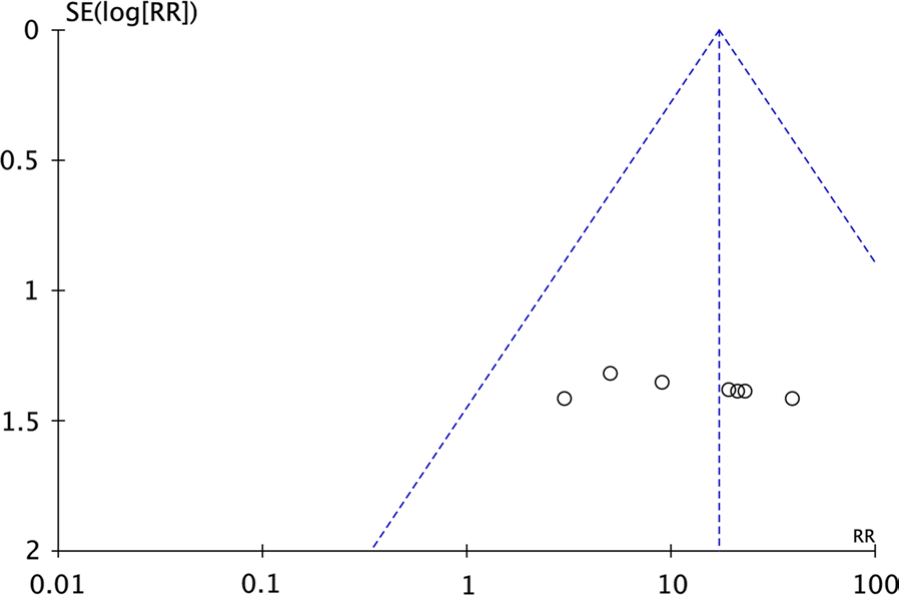
Funnel plot for changes in BCVA after SCT at month 6. The points are all within the funnel and scattered symmetrically

Seven studies [23, 33, 35, 36, 39–41] reported changes in the BCVA of the study eyes 6 months after SCT. The fixed-effects risk ratio comparing post-operative (6 months) and preoperative BCVA values was 17.00 (95% CI 6.08–47.56, *P* < 0.00001). The model had low heterogeneity (*I*^2^ = 0%). The post-operative group had favourable outcomes at 6 months (Fig. 5). In subgroup analysis using the fixed-effects model comparing BCVA in preoperative and post-operative (6 months) groups, the human embryonic stem cell-derived retinal pigment epithelium (hESC-RPE) had a risk ratio of 12.00 (95% CI 1.80–79.82, *P* = 0.01). The corresponding risk ratio of the adipose-derived stem cells (ADSCs) was 23.67 (95% CI 4.87–114.93, *P* < 0.0001), and that of the bone marrow containing CD34+ stem cells (BM with CD34+ SCs) was 12.00 (95% CI 1.75–82.06, *P* = 0.01) (Fig. 6). The funnel plot showed changes in the BCVA values at 6 months post-SCT in patients with dry AMD (Fig. 7).

**Fig. 8:**
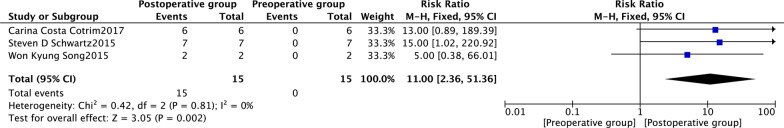
Forest plot showing the impact of SCT therapy on BCVA at month 12. The risk ratio in the fixed-effects model between post-operative and preoperative groups in BCVA after 12 months was 11.00 (95% confidence interval: 2.36–51.36, *P* = 0.002). Heterogeneity was low (*I*^2^ = 0%)

Three studies [33, 40, 41] reported changes in the BCVA of patients at 12 months after SCT. The risk ratio in the fixed-effects model comparing the BCVA values in post-operative (12 months) and preoperative groups was 11.00 (95% CI 2.36–51.36, *P* = 0.002) with low heterogeneity (*I*^2^ = 0%) (Fig. 8).

### Systemic and ocular adverse events

Four SCT-related ocular adverse events (retinal haemorrhage, oedema, focal RD, or RPE detachment [34]) were reported. No SCT-related systemic adverse event was reported. Ocular adverse events were resolved with improved intraoperative haemostasis [34].

## Discussion and limitations

No previous systematic review or meta-analysis has evaluated changes to BCVA or adverse event rates in patients with dry AMD undergoing SCT. This meta-analysis suggests that SCT may improve the BCVA values in this patient group. 

Patients with dry AMD experienced improvement to BCVA at 6 and 12 months post-SCT, compared to baseline values, suggesting STC efficacy in this context. Among-study heterogeneity was low (*I*^2^ = 0%). Subgroup analysis revealed that stem cell types, such as hESC-RPE, ADSCs, and BM with CD34+ SCs, had different BCVA outcomes at follow-up. A funnel plot revealed a low risk of publication bias for BCVA outcomes at 6 months. SCT emerged as a relatively safe procedure with few adverse events, which likely occurred due to the mode of stem cell implantation but not due to stem cell presence in the subretinal space. However, different stem cell types may require different routes of administration. This preliminary evidence suggests that SCT is a relatively safe and promising therapy for dry AMD in the short term. Further studies are required for these bioengineering techniques to be clinically available. Clinical application requires further evidence on the effects of each stem cell type, administration method, and immunosuppression strategies used in this context.

Some stem cell-based therapies are currently available in clinical trials. Patients with dry AMD received hESC-RPE transplantation, which improved their BCVA and vision-related quality-of-life [40, 41]. Although no adverse proliferation, rejection, or serious ocular or systemic safety issues have been reported [40, 41], concerns remain about the risk of teratoma formation, immune reaction, and tumorigenicity in the long term. Methods of effective immunosuppression management in hESC-RPE therapy remain unclear. Monolayers of allogeneic neonatal RPE grafts can suppress systemic delayed-type hypersensitivity when transplanted intraocularly (subretinal) through the CD95 mediated pathway [44]. This evidence suggests that hESC-RPE may be a source of tissue in subretinal administration with controlled immunosuppression. One study on hESC-RPE therapy reported that methylprednisolone administered once on day 0 immediately before surgery and tacrolimus administered from day 8 to day 60 resulted in good graft tolerability at 1-year post-implantation [34]. Immature subretinal surgery may induce retinal haemorrhage, oedema, focal RD, or RPE detachment likely due to the subretinal haemorrhage [34]. Significant perioperative haemorrhage was eliminated by not using systemic anticoagulants in the perioperative period, performing diathermy at the retinotomy site in cases of intraoperative bleeding, evacuating subretinal haemorrhage before and after hESC-RPE implantation, and elevating intraocular pressure during and after implantation [34].

One study reported that ADSCs implantation improved BCVA, visual field range, and multifocal electroretinogram findings in patients with dry AMD [23]. ADSC therapy was administered between the choroid and sclera without any immunosuppression agents likely due to the use of an autograft [23, 35, 36]. Mesenchymal stem cells (MSCs) provide nutritional support in slow retinal degeneration and immunosuppression; however, they are associated with low cell migration and differentiation rates [23]. Compared to bone marrow MSCs, adipose tissue-derived MSCs are easier to harvest from donors, grow faster, secrete more proteins, and have stronger immune regulation properties [23]. These characteristics are relevant in clinical applications. Therefore, the use of ADSCs for dry AMD in an outpatient setting is easier than the use of alternatives; in addition, it has high success rates and is associated with appropriate treatment. Meanwhile, patients with dry AMD who received the BM with CD34+ SCs therapy by an intravitreal injection experienced improved BCVA [33, 39]. However, intravitreal treatment methods, although simple and easy to perform and based on procedures used in anti-VEGF treatment, are not suitable for the introduction of cellular materials into the vitreous chamber [45]. The vitreous should remain transparent [45]. Transplantation techniques may improve with the direct use of the mesenchymal secretome and its active components, which may help prevent complications related to the intravitreal injection of cells or those related to procedures that require interventions under the retina. Although iPSCs-RPE therapy was not included in the meta-analysis, it remains a relevant approach. One study has shown that iPSCs-RPE therapy is more effective than MSC and neural stem cell therapies at delaying photoreceptor cell loss after implantation into the subretinal space in *rd1* mice with progressive retinal degeneration [46]. Further studies can be conducted to compare the effects of different stem cell types in clinical studies.

Overall, mature SCT may be a suitable treatment method for dry AMD, improving BCVA outcomes of affected patients. Other patient groups may benefit from this treatment, including patients with retinal degeneration, such as diabetic retinopathy, RP, and Stargardt’s macular degeneration. However, further evidence and strict guidelines are required for successful implementation. Future studies should explore SCT mechanisms, compare stem cell type efficacy and safety, and test stem cell-derived exosomes in the treatment of retinal degeneration. In addition, long-term effects, administration technique, and approaches to personalised treatment require further research [4, 47].

This study had some limitations. First, because of the un-synthetic reasons, existing clinical data detected by microperimetry, fundus autofluorescence, spectral domain-optical coherence tomography (SD-OCT), and ERG are not applied to the meta-analysis. Second, this meta-analysis did not compare outcomes among SCT types. Third, associated ocular and systemic adverse event reports were incomplete and the estimates varied, suggesting that further studies are required to assess SCT safety. Fourth, long-term outcomes were not assessed in this study due to the lack of data. Fifth, this meta-analysis included few studies, which had small sample sizes, increasing the risk of bias.

## Conclusion

This meta-analysis of fewer randomised controlled trials suggests that SCT may help improve BCVA in patients with dry AMD.

## Supplementary Information

**Additional file 1.** We searched the articles according to the results of Mesh words and Free words.

## Data Availability

Data sharing is not applicable to this article as no datasets were generated or analysed during the current study.

## Abbreviations

AMD: Age-related macular degeneration
ADSCs: Adipose-derived stem cells
BCVA: Best-corrected visual acuity
BM with CD34+ SCs: Bone marrow containing CD34+ stem cells
CI: Confidence interval
hESC-RPE: Human embryonic stem cell-derived retinal pigment epithelium
iPSCs-RPE: Induced pluripotent stem cells-derived retinal pigment epithelium
MSCs: Mesenchymal stem cells
RR: Risk ratio
RPE: Retinal pigment epithelium
RP: Retinitis pigmentosa
RD: Retinal detachment
SCT: Stem cell transplantation
SMD: Stargardt’s macular degeneration
SD-OCT: Spectral domain-optical coherence tomography
VEGF: Vascular endothelial growth factor
