# Supplementary material for: Changes in best-corrected visual acuity in patients with dry age-related macular degeneration after stem cell transplantation: systematic review and meta-analysis
Source: Stem Cell Res Ther. 2022 Jun 7;13:237. doi: 10.1186/s13287-022-02931-y (PMC9172101; doi:10.1186/s13287-022-02931-y)
Supplement: Supplementary file 1 — Additional file 1. We searched the articles according to the results of Mesh words and Free words. [file 13287_2022_2931_MOESM1_ESM.doc]

**Additional file 1 Results of Mesh words AND Free words.**

| Database | Mesh words AND Free words | Results |
| --- | --- | --- |
| PubMed | ("macular degeneration"[MeSH Terms] OR "degeneration macular"[Title/Abstract] OR "macular degenerations"[Title/Abstract] OR "Maculopathy"[Title/Abstract] OR "Maculopathies"[Title/Abstract] OR "macular dystrophy"[Title/Abstract] OR "dystrophy macular"[Title/Abstract] OR "macular dystrophies"[Title/Abstract] OR "age related macular degeneration"[Title/Abstract] OR "age related macular degeneration"[Title/Abstract] OR "age related macular degenerations"[Title/Abstract] OR "macular degeneration age related"[Title/Abstract] OR "macular degeneration age related"[Title/Abstract] OR "maculopathies age related"[Title/Abstract] OR "maculopathy age related"[Title/Abstract] OR "maculopathy age related"[Title/Abstract] OR "age related maculopathies"[Title/Abstract] OR "age related maculopathies"[Title/Abstract] OR "age related maculopathy"[Title/Abstract] OR "age related maculopathy"[Title/Abstract]) AND ("stem cell transplantation"[MeSH Terms] OR "stem cell transplantations"[Title/Abstract] OR "transplantations stem cell"[Title/Abstract] OR "transplantation stem cell"[Title/Abstract]) | 210 |
| EMBASE | ('macular degeneration'/exp OR 'degeneration, macular':ab,ti OR 'macular degenerations':ab,ti OR maculopathy:ab,ti OR maculopathies:ab,ti OR 'macular dystrophy':ab,ti OR 'dystrophy, macular':ab,ti OR 'macular dystrophies':ab,ti OR 'age-related macular degeneration':ab,ti OR 'age related macular degeneration':ab,ti OR 'age-related macular degenerations':ab,ti OR 'macular degeneration, age-related':ab,ti OR 'macular degeneration, age related':ab,ti OR 'maculopathies, age-related':ab,ti OR 'maculopathy, age-related':ab,ti OR 'maculopathy, age related':ab,ti OR 'age-related maculopathies':ab,ti OR 'age related maculopathies':ab,ti OR 'age-related maculopathy':ab,ti OR 'age related maculopathy':ab,ti) AND ('stem cell transplantation'/exp OR 'stem cell transplantations':ab,ti OR 'transplantations, stem cell':ab,ti OR 'transplantation, stem cell':ab,ti) | 587 |
| Cochrane | ((MeSH descriptor: [Macular Degeneration] explode all trees) OR (Degeneration, Macular):ti,ab,kw OR (Macular Degenerations):ti,ab,kw OR (Maculopathy):ti,ab,kw OR (Maculopathies):ti,ab,kw OR (Macular Dystrophy):ti,ab,kw OR (Dystrophy, Macular):ti,ab,kw OR (Macular Dystrophies):ti,ab,kw OR (Age-Related Macular Degeneration):ti,ab,kw OR (Age Related Macular Degeneration):ti,ab,kw OR (Age-Related Macular Degenerations):ti,ab,kw OR (Macular Degeneration, Age-Related):ti,ab,kw OR (Macular Degeneration, Age Related):ti,ab,kw OR (Maculopathies, Age-Related):ti,ab,kw OR (Maculopathy, Age-Related):ti,ab,kw OR (Maculopathy, Age Related):ti,ab,kw OR (Age-Related Maculopathies):ti,ab,kw OR (Age Related Maculopathies):ti,ab,kw OR (Age-Related Maculopathy):ti,ab,kw OR (Age Related Maculopathy):ti,ab,kw) AND ((MeSH descriptor: [Stem Cell Transplantation] explode all trees) OR (Stem Cell Transplantations):ti,ab,kw OR (Transplantations, Stem Cell):ti,ab,kw OR (Transplantation, Stem Cell):ti,ab,kw) | 13 |
